# Convection enhanced delivery of panobinostat (LBH589)-loaded pluronic nano-micelles prolongs survival in the F98 rat glioma model

**DOI:** 10.2147/IJN.S125300

**Published:** 2017-02-21

**Authors:** WG Singleton, AM Collins, AS Bienemann, CL Killick-Cole, HR Haynes, DJ Asby, CP Butts, MJ Wyatt, NU Barua, SS Gill

**Affiliations:** 1 Functional Neurosurgery Research Group, School of Clinical Sciences, University of Bristol; 2 Department of Neurosurgery, North Bristol NHS Trust; 3 Bristol Centre for Functional Nanomaterials, School of Physics, HH Wills Physics Laboratory; 4 Brain Tumour Research Group, School of Clinical Sciences; 5 School of Chemistry, University of Bristol, Bristol, UK

**Keywords:** glioma, micelle, convection enhanced delivery, panobinostat, HDAC inhibitor

## Abstract

**Background:**

The pan-histone deacetylase inhibitor panobinostat is a potential therapy for malignant glioma, but it is water insoluble and does not cross the blood–brain barrier when administered systemically. In this article, we describe the in vitro and in vivo efficacy of a novel water-soluble nano-micellar formulation of panobinostat designed for administration by convection enhanced delivery (CED).

**Materials and methods:**

The in vitro efficacy of panobinostat-loaded nano-micelles against rat F98, human U87-MG and M059K glioma cells and against patient-derived glioma stem cells was measured using a cell viability assay. Nano-micelle distribution in rat brain was analyzed following acute CED using rhodamine-labeled nano-micelles, and toxicity was assayed using immunofluorescent microscopy and synaptophysin enzyme-linked immunosorbent assay. We compared the survival of the bioluminescent syngenic F98/Fischer344 rat glioblastoma model treated by acute CED of panobinostat-loaded nano-micelles with that of untreated and vehicle-only-treated controls.

**Results:**

Nano-micellar panobinostat is cytotoxic to rat and human glioma cells in vitro in a dose-dependent manner following short-time exposure to drug. Fluorescent rhodamine-labelled nano-micelles distribute with a volume of infusion/volume of distribution (Vi/Vd) ratio of four and five respectively after administration by CED. Administration was not associated with any toxicity when compared to controls. CED of panobinostat-loaded nano-micelles was associated with significantly improved survival when compared to controls (n=8 per group; log-rank test, *P*<0.001). One hundred percent of treated animals survived the 60-day experimental period and had tumour response on post-mortem histological examination.

**Conclusion:**

CED of nano-micellar panobinostat represents a potential novel therapeutic option for malignant glioma and warrants translation into the clinic.

## Introduction

High-grade glioma (HGG) is the most common malignant primary brain tumor in adults, and its treatment represents an unmet clinical need in both children and adults. The age-specific incidence of HGG is bimodal, peaking in childhood and to a greater extent between 50 and 60 years of age. Despite current treatment involving a combination of surgery, systemic chemotherapy and radiotherapy, the prognosis of glioblastoma (GBM) is poor with a median survival of 14.6 months in adults.^1^ Many promising therapeutic agents for central nervous system (CNS) disorders have failed to attain clinical success due to the blood–brain barrier (BBB), which prevents the passage of agents from the systemic circulation into the brain. Systemic administration of high drug doses may increase delivery to the brain, but this approach risks significant side effects and toxicity. Direct delivery of drugs to the brain facilitates bypass of the BBB. However, the therapeutic efficacy of drugs injected into the brain parenchyma and/or tumors is limited by minimal diffusion from the site of injection and, consequently, small volumes of distribution. In 1994, the concept of convection enhanced delivery (CED) was introduced as a solution to the obstacles that limit therapeutic drug delivery to the CNS.^2^,^3^

CED describes continuous infusion of agents under pressure through stereotactically placed micro-catheters.^4^ This method has several potential advantages over conventional drug delivery methods. CED facilitates highly accurate anatomical drug targeting, delivery of high drug concentrations throughout clinically relevant volumes of brain tissue or tumor, and reduces systemic side effects. CED has been extensively investigated in the context of a wide range of CNS disorders in both preclinical and clinical trials, most notably for the treatment of brain tumors and Parkinson’s disease.^5^–^11^ Drugs can be administered directly to the brain by CED in concentrations that would result in significant toxicity if given systemically. In contrast to delivery techniques that are dependent on diffusion, CED enables the controlled, homogeneous distribution of drugs through large brain volumes.^3^ Furthermore, as CED leads to the displacement of extracellular fluid with infusate, it offers an opportunity to manipulate the extracellular environment of intrinsic malignant brain tumors.^12^–^15^

Panobinostat is a pan-histone deacetylase inhibitor (HDACi), which has recently gained the approval of the US Food and Drug Administration (FDA) and National Institute for Health and Care Excellence UK for the treatment of relapsed myeloma in adults. Histone deacetylation results in chromatin remodeling and controls a number of cellular processes, including the regulation of transcription, transcription factor stability and cell survival. Inhibition of this histone deacetylation results in cell cycle arrest and apoptosis of tumor cells.^16^ Epigenetic events, such as histone acetylation, are believed to be critical processes that drive gliomagenesis in both adult and pediatric supratentorial HGG and in pediatric brainstem glioma, including diffuse intrinsic pontine glioma (DIPG).^17^,^18^ A recent drug screen of genetically diverse and clinically relevant ex vivo DIPG cell lines revealed panobinostat as the most efficacious drug currently in clinical use.^19^ Panobinostat therefore may be a particularly suitable drug candidate for both supratentorial and brainstem HGG chemotherapy in both adults and children.

Oral panobinostat has been studied in phase 1 and 2 clinical trials in adult patients with progressive GBM. The phase 1 results describe a maximum tolerated dose of 30 mg three times per week, every other week when given in combination with bevacizumab, an anti-vascular endothelial growth factor monoclonal antibody.^20^ The phase 2 trial was stopped prematurely as it did not show a significant survival benefit when compared to control.^21^ In a recent clinical study of patients treated with panobinostat as a method of reactivating latent HIV to a therapeutic advantage, panobinostat was not detectable in the cerebrospinal fluid (CSF) at multiple time points post treatment, suggesting that treatment failure in patients with HGG may be due to poor BBB penetrance.^22^ A similar result has been reported in phase 1 data from children receiving oral panobinostat for the treatment of acute lymphoblastic leukemia and from nonhuman primate pharmacokinetic data.^23^,^24^ Direct intratumoral infusion of panobinostat by CED is therefore of significant clinical interest for the treatment of supratentorial and brainstem HGG.

Panobinostat is very poorly water soluble (<1 mg/mL). The physicochemical characteristics and hydrophobicity of panobinostat make it an unlikely candidate for CED in its unmodified form.^25^ Chemical modification of panobinostat to achieve water solubility risks negation of its biochemical efficacy. To overcome these limitations, we investigated the delivery of panobinostat as a payload in nano-micellar form. A number of carrier vehicles exist that are capable of sequestering a strongly hydrophobic compound within the core of a particle measuring several tens of nanometers in diameter, which are dispersible in water.^26^ It is possible for these particulates to disseminate through the body and be taken up by cells, whereupon the drug payload is delivered by cellular mechanisms to the target site, facilitating bypass of the major efflux transporters of the BBB.^27^

Poloxamer 407 (P407) is an FDA-approved triblock copolymer consisting of hydrophobic polypropylene glycol units terminated at each end by hydrophilic polyethylene glycol chains. The hydrophobic and hydrophilic interactions within the P407 molecule drive self-assembly into micelles ~20 nm in diameter in aqueous solutions.^28^ It is possible for strongly hydrophobic drugs to be partitioned within the micelle core, such that they can be dispersed under physiological conditions as a colloid. Compounds that cannot be directly solubilized into the hydrophobic core through mixing alone can be formulated into particles by an emulsion-mediated solvent evaporation method.^29^,^30^ This mechanism achieves drug concentrations that are far greater than normal for a species dissolved into aqueous physiological solutions. This has been previously demonstrated by loading polymeric nano-micelles with the organometallic drug KP46.^30^

We describe a series of experiments that demonstrate the in vitro efficacy of water-soluble panobinostat-loaded P407 nano-micelles (LBH589/P407) against commercially available and patient-derived HGG cell lines, their distribution and toxicity after CED in normal rat brain, and the effect on survival when administered to the orthotopic F98 rat glioma model by CED.

## Materials and methods

### Micelle synthesis and evaluation

#### Formulation of drug-loaded polymeric nano-micelles in artificial cerebrospinal fluid (aCSF)

A stock solution of 5% P407 in aCSF (Torbay Pharmaceuticals, Torbay, UK) was prepared by the direct dissolution of P407 polymer into aCSF under magnetic stirring. Control solutions of unloaded polymeric nano-micelles were prepared by the addition of 0.5 mL of CHCl_3_ to 10 mL of a 5% P407 in aCSF solution (P407/aCSF) under stirring to form an emulsion. After a minimum of 15 minutes of stirring, the emulsion solution was transferred to a beaker and microwaved in ten seconds bursts with manual stirring until the CHCl_3_ was observed to evaporate. The aqueous nano-micelle product was then extracted from the beaker in a graduated syringe and passed through a 250-μm filter into a vial and then sealed. The critical micelle concentration for P407 at 25°C in water is 0.04%.^31^ This is much lower than the 5% used in making the stock solution in aCSF. We found that lower concentrations of P407 during the drug-loading step failed to support the drug in solution.

Drug-loaded polymeric nano-micelle solutions were formed using the same process but with a given amount of panobinostat dissolved in the organic phase prior to addition to the aqueous phase. Stock of 0.5 mL (10 mg/mL) panobinostat (molecular weight [MW] =349.4; SelleckChem, Munich, Germany) in chloroform (CHCl_3_) was added to 10 mL of stock P407/aCSF and emulsified before microwave treatment and filtration to produce a colloid with the equivalent concentration of 0.5 mg/mL panobinostat. Nano-micelle solutions were prepared at lower loadings of drug by dilution of the 10 mg/mL stock chloroform solution before addition to the aqueous phase. Panobinostat-loaded P407 nano-micelles in aCSF solutions (LBH589/P407) were prepared in a drug concentration range of 0.083–0.5 mg/mL.

#### Characterization of P407 nano-micelles

The size and charge of unloaded control and drug-loaded P407 nano-micelles were analyzed by dynamic light scattering (DLS) and zeta potential measurement using a Zetasizer Nano ZS (Malvern Instruments).

^1^H nuclear magnetic resonance (NMR) experiments to investigate the behavior of the drug-loaded polymeric nano-micelles over time and upon dilution were conducted on a Varian 600 MHz VNMRS NMR Spectrometer fitted with a 5-mm HCN cold probe and using a double pulse field-gradient spin-echo water suppression pulse sequence.

^1^H diffusion-ordered NMR spectroscopy (DOSY NMR) was performed on a Varian 500 MHz VNMRS NMR Spectrometer fitted with a 5-mm AutoX DB-PFG probe to confirm the presence and diffusion coefficient of free panobinostat. Full sample preparation for NMR experiments is available in the Supplementary materials.

#### Synthesis of rhodamine-labeled P407 nano-micelles

An amino-terminated P407 intermediate (P407-NH_2_) was synthesized and subsequently functionalized further with rhodamine through imidazole cross-linking using a method adapted from those previously described.^32^,^33^ For micelle-labeling experiments, 0.05% of rhodamine-labeled P407 to unlabeled P407/aCSF was determined to be optimal for fluorescent-imaging purposes.

### In vitro experiments

#### Cell culture

F98, U87-MG and M059K glioma cell lines (American Type Culture Collection, ATCC, Manassas, VA, USA) were grown following the supplier’s protocol and under standard conditions. In brief, U87-MG and F98 were cultured in Dulbecco’s Modified Eagle’s Medium (DMEM) supplemented with 10% fetal bovine serum and 1% l-glutamine, whereas M059K cells were grown in DMEM/Ham-F12, with 10% fetal bovine serum, 1.5% HEPES, 1% l-glutamine and 1% nonessential amino acids at 37°C in a humidified atmosphere with 5% CO_2_. Reagents were purchased from Life Technologies (Paisley, UK) unless stated otherwise. Patient-derived glioma stem cell lines, G144 and G26 were obtained as a kind gift from Dr Colin Watts (University of Cambridge, Cambridge, UK) and Dr Steven Pollard (University of Edinburgh, Edinburgh, UK) and cultured following instructions from the host laboratories.^34^,^35^

#### Primary hippocampal cell culture

Neuronal cultures were obtained from the hippocampus of 18-day-old Wistar rat embryos as previously described.^36^ Cells were counted and plated on poly-D-lysine-coated 10 mm coverslips (Sigma-Aldrich, Dorset, UK) at a density of 75,000 cells per coverslip.

#### In vitro cell viability assay

Cellular cytotoxicity of LBH589/P407 nano-micelles was measured using a two-color fluorescent-based live/dead cell viability assay (Life Technologies). Cells were cultured in 96-well plates and dosed with LBH589/P407 for 30 minutes, 6 hours and 72 hours. After 72 hours, cells were washed with phosphate-buffered saline (PBS) and incubated with 2 μM calcein acetoxymethyl and 4 μM ethidium homodimer-1 for 30 minutes at room temperature. Fluorescence was measured on a microplate reader (FLUOstar Omega; BMG Labtech), following the manufacturer’s protocol. All assays were performed in triplicate using cells of three different passages. Experimental values were normalized against negative and positive controls.

#### Generation of F98-luciferase-expressing cells

To create a bioluminescent glioma cell line, F98 rat glioma cells were transduced with firefly luciferase at a multiplicity of infection of 10 for 18 hours as described by the manufacturer (Cignal Lenti [Luc]; Qiagen, Manchester, UK), followed by puromycin selection.

### In vivo experiments

#### CED procedure

Juvenile Wistar rats (225–275 g; Charles River, Margate, UK) were housed in the animal service unit facility at the University of Bristol. All animal-handling procedures and experiments were performed in accordance with the UK Animal Scientific Procedures Act 1986 and covered by UK Home Office licenses approved by the University of Bristol ethics committee and institutional review board (project licence: 30/2909).

Animals were anesthetized with intraperitoneal ketamine (Ketaset, 60 mg/kg; Pfizer Animal Health, Sandwich, UK) and medetomidine (Dormitor, 0.4 mg/kg; Pfizer Animal Health), and then placed in a stereotactic frame (David Kopf Instruments, Tujunga, CA, USA). A midline skin incision was made from glabella to occiput to expose bregma. CED procedures were performed using a custom-made catheter with an outer diameter of 0.22 mm and inner diameter of 0.15 mm, composed of fused silica with a laser cut tip. The cannula was attached to a 1 mL syringe (Hamilton, Bonaduz, Switzerland) connected to a rate-controlled micro-infusion pump (World Precision Instruments Inc., Sarasota, FL, USA) and the tip placed at stereotactic coordinates derived from the Paxinos and Watson stereotactic rat brain atlas (0.75 mm rostral and 3 mm lateral to bregma, at a depth of 5 mm to target the striatum, and 2.5 mm to target the corpus callosum). Burr holes were drilled using a 2-mm drill.

All CED procedures were performed at an infusion rate of 2.5 μL/minute. On completion of infusion, the cannula was left in situ for 10 minutes to minimize reflux, and then it was withdrawn at a rate of 1 mm/minute. The wound was closed, and a dose of intramuscular buprenorphine (Centaur Services, Castle Cary, UK) was administered (30 μg/kg). The anesthetic was reversed with 0.1 mg/kg intraperitoneal atipamezole hydrochloride (Pfizer Animal Health) in recovery procedures. At predetermined time points, animals were euthanized by anesthetic overdose with an intraperitoneal injection of 1 mL pentobarbital (Euthatal; Merial Animal Health, Harlow, UK). Then, either perfusion fixed with 100 mL of PBS followed by 100 mL of 4% paraformaldehyde (PFA; Fisher Scientific, Loughborough, UK) in PBS (pH 7.4) or the brain was removed from the skull and placed on dry ice immediately for further analysis. If perfusion fixed, the brain was then removed from the skull, placed in 4% PFA for 48 hours and cryoprotected in 30% sucrose (Melford Laboratories, Ipswich, UK) in PBS prior to sectioning.

#### Distribution analysis of rhodamine-labeled P407 nano-micelles

Fluorescent rhodamine-labeled P407 nano-micelles were infused into the striatum and white matter of Wistar rats for distribution analysis. Two groups of three animals were sacrificed at 0 and 48 hours post infusion.

Rat brains were then cut into 35 μm thick coronal sections using a Leica CM1850 cryostat (Leica Microsystems, Wetzlar, Germany) at −20°C and mounted on gelatin-subbed slides. Images were captured using the Stereo Investigator platform (MicroBrightField Bioscience, Williston, VT, USA) using a Leica DM5500 microscope (Leica Microsystems) and digital camera (MicroBrightField Bioscience). Images were analyzed with in-house software, which calculates the three-dimensional (3D) volume from automated segmentation of fluorescent signal from serial two-dimensional (2D) images.

### In vivo toxicity analysis

Toxicity was determined using quantitative analysis of the presynaptic protein synaptophysin and postmortem immunofluorescent microscopy after acute striatal infusion of unloaded P407 nano-micelles (P407/aCSF) in the left striatum and aCSF in the right striatum to serve as a control. Juvenile Wistar rats were assigned to experimental groups and sacrificed at 48 hours or 21 days in two groups of three animals for histological examination or synaptophysin enzyme-linked immunosorbent assay (ELISA).

### Synaptophysin ELISA

Rats were euthanized by anesthetic overdose as described earlier, and their brains were removed and snap-frozen in liquid nitrogen. Synaptophysin ELISA was performed on rat brain homogenates at 48 hours and 21 days following CED of P407 aCSF or aCSF alone. An untreated age-matched control brain was used as an additional control. The ELISA protocol for measuring synaptophysin concentration was adapted for rat brain homogenates as previously described.^37^,^38^ All samples were tested in triplicate.

### Immunofluorescent microscopy

Thirty five micrometer thick sections of PFA-fixed rat brain were mounted on gelatin-subbed slides. The sections were incubated with either mouse anti-rat NeuN, clone A60, biotin-conjugated (1:100; Millipore, Temecula, CA, USA) or rabbit anti-rat GFAP (1:300; Millipore) primary antibodies overnight at 4°C. Detection was determined using donkey anti-mouse (1:150; streptavidin Alexa Fluor 488; Jackson Laboratories, Sacramento, CA, USA) or donkey antirabbit (1:300; Cy3; Jackson Laboratories). Sections were also treated with DAPI (1:200 of 1 mg/mL; Sigma-Aldrich) prior to mounting in FluorSave™ reagent (Calbiochem; Merck Millipore, Billerica, MA, USA).

### F98 glioma model and survival study design

An F98 cellular suspension containing 100,000 luciferase-expressing cells was implanted into the left striatum of 24 Fischer-344 rats using the methods previously described.^39^ Post operative recovery was the same as described earlier for animals undergoing CED procedures.

Animals were placed under brief isoflurane inhalation anesthetic and underwent transcranial bioluminescent imaging at weekly intervals after tumor cell implantation. Each animal had 150 mg/kg intraperitoneal luciferin (StayBrite™; BioVision Inc., Milpitas, CA, USA) injected 30 minutes prior to imaging. Animals that had no visible transcranial bioluminescence 1 week after tumor implantation were excluded from the study.

A total of 24 animals were randomly assigned to three treatment groups each consisting of eight animals: group 1, treatment (0.6 μg/mL LBH589/P407); group 2, vehicle-only treated control (P407/aCSF); and group 3, untreated control. Animals in the two treatment arms underwent CED of either vehicle or drug 10 days after tumor implantation. The CED procedure was identical to that described earlier and used the same burr hole created for tumor implantation. Animals were examined daily and underwent bioluminescent imaging every 7 days by personnel who were blinded to the groups. Animals that had lost 10% of body weight or showed signs of neurological disability were terminated by schedule 1 killing, underwent transcardial 4% PFA perfusion, brain explantation and brain preservation in 4% PFA prior to pathological examination. The experiment continued for 60 days when all surviving animals were terminated.

### Postmortem histology

Rat brains were processed on a Leica Peloris and embedded in paraffin. Brains were sectioned at 3 μm thickness and stained with hematoxylin and eosin (H&E), using a Leica Autostainer XL as per standard histopathological technique. All pathological samples were examined by a neuropathologist who was blinded to the experimental groups.

### Data and statistical analysis

All data analysis was performed using GraphPad Prism^®^ v5 (GraphPad Software Inc., San Diego, CA, USA). IC_50_ values were calculated from logarithmic dose–response curves. Multiple groups of data were compared using one-way analysis of variance. Significance was calculated using Bonferroni’s multiple comparison post hoc analysis. Survival curves were plotted using the Kaplan–Meier method, and significance was calculated using log-rank test. Significance was defined as *P<0.05, **P<0.01 and ***P<0.001.

## Results

### Synthesis and characterization of panobinostat-loaded nano-micelles

Colloids of panobinostat stabilized by 5% P407 in aCSF were successfully prepared by an emulsion-mediated process of organic cosolvent evaporation. The addition of a chloroform solution of panobinostat to P407/aCSF under stirring produced an opaque white emulsion that turned to a transparent clear solution after microwave heating and removal of the chloroform phase. DLS of a control solution of P407/aCSF gave an average micelle diameter of 23 nm. DLS measurements of LBH589/P407-loaded nano-micelles across a concentration range gave an average micelle diameter of 26 nm (Figure S1). Both unloaded and drug-loaded P407 nano-micelles were found to have a neutral surface charge by zeta potential measurement.

**Figure 1:**
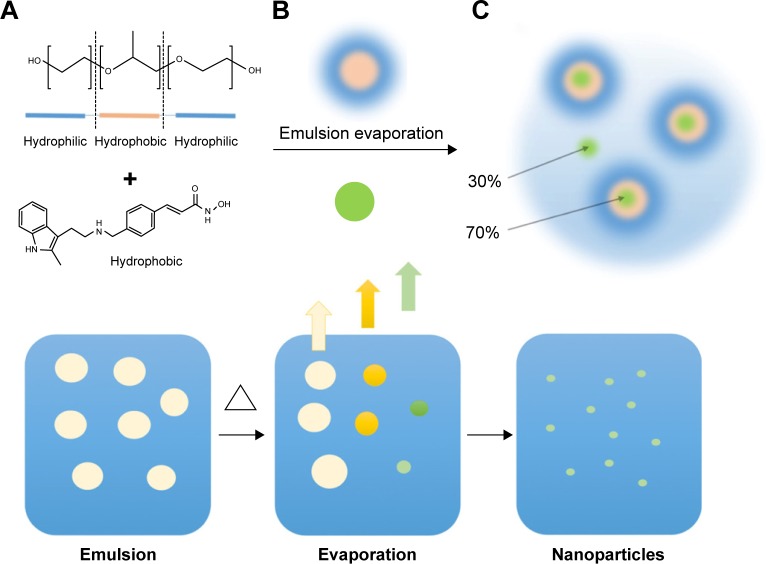
A schematic representation of the emulsion-mediated solvent evaporation preparation method. **Notes:** (**A**) A solution of P407 (orange and blue) in aCSF is emulsified with a solution of panobinostat in chloroform. (**B**) The emulsion is heated to rapidly remove the chloroform and to sequester panobinostat (green) in the polymeric nano-micelles. A schematic representation of this process is shown in the bottom half of the figure. On the left is an emulsion of CHCl_3_ containing the drug payload and stabilized by the P407 surfactant. The emulsion is heated, and the CHCl_3_ evaporates to leave P407 nano-micelles containing the drug payload represented on the right. (**C**) After evaporation, ~70% is loaded into the nano-micelles while 30% is present as a solvated molecular species. **Abbreviations:** aCSF, artificial cerebrospinal fluid; P407, poloxamer 407.

NMR spectroscopy can detect the presence of panobinostat free in solution. Drug present as either an insoluble portion or sequestered within the core of a polymeric micelle is not detectable. NMR spectroscopy of LBH589/P407 formulations will indicate any dynamic equilibrium and release of the drug from the micelles upon further dilution in aCSF. NMR spectra of LBH589/P407 colloids at neat (0.5 mg/mL), 1:3 and 1:6 dilutions in both aCSF and P407/aCSF were taken. In a 400 μL sample, 60 μg LBH589 was detectable, representing the presence of 30% of the drug as a molecular species free in solution, with 70% undetectable within the cores of polymeric micelles (Figure 1). Upon dilution of 0.5 mg/mL LBH589/P407, to a third of initial concentrations with both aCSF and P407/aCSF, the LBH589 NMR signal integral also dropped by a third. No further release of LBH589 into solution was observed after 24 hours. A similar observation was seen when fresh samples were diluted to a sixth of their initial concentrations with pure aCSF or P407/aCSF. For comparative purposes, three 200 μg LBH589 samples were each added to pure water, pure aCSF and a P407/aCSF solution, respectively, and agitated over 24 hours in an attempt to get the drug to dissolve directly. For each, up to 10 μg was observable as a solubilized species by NMR, indicating that the emulsion-mediated solvent evaporation mechanism is critical in achieving a high loading of drug in solution.

^1^H DOSY NMR was used to determine the diffusion coefficients of the solvated panobinostat molecular portion. A control solution of panobinostat in dimethyl sulfoxide yielded a diffusion coefficient of 1–2×10^−10^ m^2^/s, consistent with values expected for a molecule free in solution. The diffusion coefficient measured for the panobinostat in a LBH589/P407 colloid was found to be 0.8–2×1^−10^ m^2^/s, suggesting that the solvated molecular panobinostat present was not associated with the P407 polymer. The calculated diffusion coefficient for a 23-nm-diameter particle using the Stokes–Einstein equation was 2.8×10^−11^ m^2^/s, an order of magnitude lower than the NMR-observed molecular species. These results indicate that two separate forms of panobinostat were present in the nano-micellar formulation having potentially different pharmacokinetic profiles (Figures S2 and S3).

### In vitro cytotoxicity of panobinostat-loaded P407 nano-micelles

**Figure 2:**
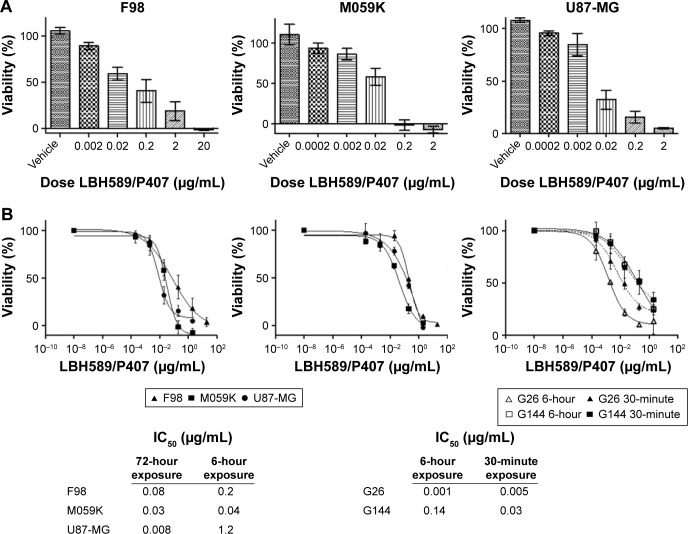
In vitro cytotoxicity of LBH589/P407 nano-micelles. **Notes:** (**A**) Dose–response effect on cell viability following 72 hours of incubation with F98, U87-MG and M059K glioma cell lines. Vehicle alone was not cytotoxic. (**B**) Logarithmic dose–response curves of LBH589/P407 nano-micelles after 72 and 6 hours of incubation with F98, U87-MG and M059K glioma cell lines. Dose–response curves of LBH589/P407 nano-micelles after 6 hours and 30 minutes of incubation with G144 and G26 patient-derived glioma stem cell lines. Tables show representative IC_50_ values for each condition. All the experiments were performed in triplicate. **Abbreviations:** P407, poloxamer 407; DAPI, 4,6-Diamidino-2-phenylindole, dihydrochloride.

**Figure 3:**
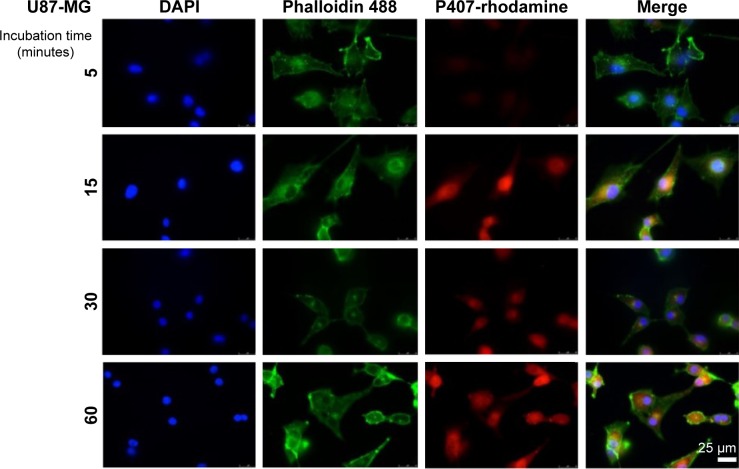
Immunofluorescent microscopy of fixed U87-MG cells after exposure to fluorescent P407-rhodamine nano-micelles for 5, 15, 30 and 60 minutes. Scale bar = 25 μm.

The rat glioma cell line, F98, and human glioma cell lines, M059K and U87-MG, were incubated with LBH589/P407 nano-micelles at increasing concentrations for 72 hours. A cell viability assay showed a dose-dependent effect. Nondrug-loaded P407/aCSF nano-micelles were not cytotoxic (Figure 2). The same assay was performed 72 hours after a 6-hour exposure to LBH589/P407, to attempt to mimic the short tumor exposure times to the drug achieved after CED in vivo. Interestingly, the dose–response curves were similar to those observed when cells had been in contact with drug for longer time periods (Figures 2 and 3).

Given that the LBH589/P407 dose–response with a 6-hour exposure time is comparable to a 72-hour exposure, we next investigated the cellular localization of P407 following increasing periods of incubation with glioma cells. Nondrug-loaded fluorescent rhodamine-labeled P407/aCSF nano-micelles were incubated with live cells (U87-MG) in culture prepared for microscopy to determine the intracellular distribution of the polymer. The fluorescent polymer was visible within the cytoplasm of the cell within 15 minutes, but not after 5 minutes of incubation and seemed to localize to the nucleus (Figure 2). This observation led us to hypothesize that even shorter incubation times with LBH589/P407 may be cytotoxic to glioma cells in vitro. LBH589/P407 had a dose–response effect on cell viability of the glioma stem cells, G26 and G144, following 30 minutes of incubation, which was comparable to the effect seen after 6 hours (Figure 2).

### In vivo distribution of rhodamine-labeled P407-rhodamine nano-micelles in a rat model of acute CED

**Figure 4:**
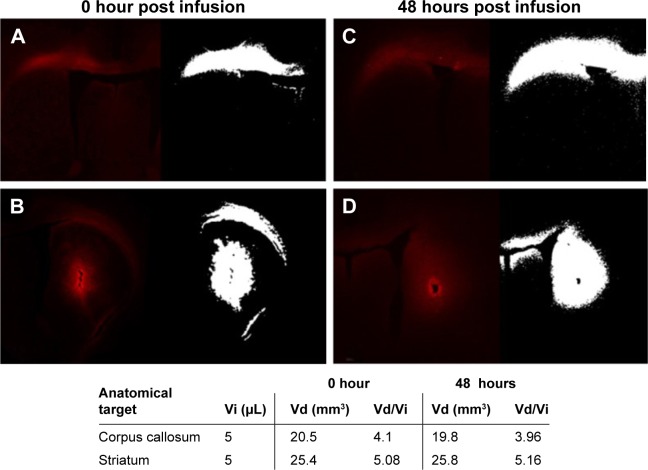
Distribution of P407-rhodamine nano-micelles after CED into rat corpus callosum (white matter) and striatum (gray matter). **Notes:** (**A**) White matter distribution 0 hour after CED. (**B**) Gray matter distribution 0 hour after CED. (**C**) White matter distribution 48 hours after CED. (**D**) Gray matter distribution 48 hours after CED. Volume of infusion (Vi) is calculated by auto-segmentation of fluorescent signal (white image) from images from adjacent 35 μm brain sections using bespoke in-house software. Volume of distribution to volume of infusion ratio (Vd/Vi) indicates bulk flow of micelles via CED rather than simple injection. **Abbreviations:** CED, convection enhanced delivery; P407, poloxamer 407.

We investigated the distribution of P407/aCSF in normal rat gray and white matter after acute CED. Rhodamine-labeled P407/aCSF was administered by CED to the corpus callosum and striatum of Wistar rats, which were sacrificed at 0 and 48 hours post infusion. Fluorescent microscopy of sections from these brains was used to calculate the volume of infusion in both white matter (corpus callosum) and gray matter (striatum). Fluorescence was detectable at both time points. The volume of distribution (Vd) in white and gray matter at both 0 and 48 hours was four to five times greater than the volume of infusion (Vi; Figure 4).

### In vitro and in vivo toxicity of P407 nano-micelles delivered by acute CED

**Figure 5:**
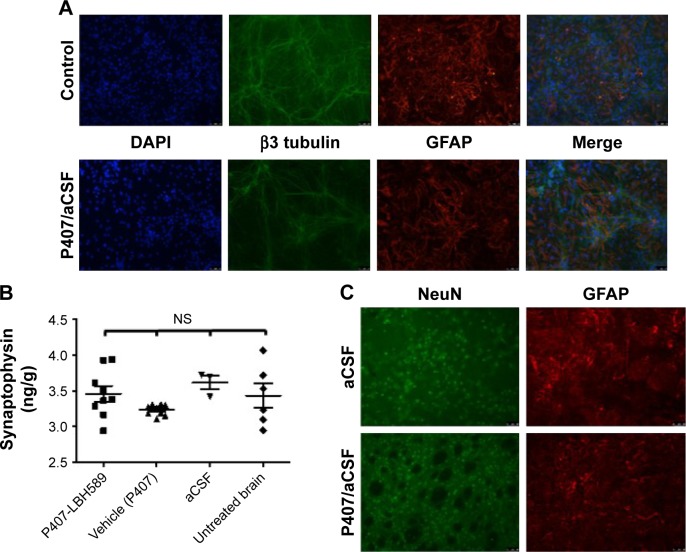
Toxicity of P407 nano-micelles. **Notes:** (**A**) In vitro morphology of a mixed primary rat glio-neuronal culture after 72 hours of incubation with 5% P407 in aCSF relative to control. (**B**) Synaptophysin ELISA of protein isolated from rat brain 21 days after infusion of either aCSF or P407/aCSF nano-micelles and from untreated tumor-bearing animals (one-way ANOVA Bonferroni’s multiple comparison test, *P*>0.05). (**C**) Immunofluorescent microscopy of fixed rat brain for neurons (NeuN) and glia (GFAP) 21 days after acute striatal infusion of either aCSF or P407/aCSF nano-micelles. **Abbreviations:** aCSF, artificial cerebrospinal fluid; ANOVA, analysis of variance; DAPI, 4,6-Diamidino-2-phenylindole, dihydrochloride; ELISA, enzyme-linked immunosorbent assay; NS, not significant; P407, poloxamer 407.

P407/aCSF nano-micelles were incubated with a primary rat hippocampal mixed glio-neuronal culture for 72 hours and were not associated with any alteration in cellular density or morphology when compared to control (Figure 5). To investigate the potential neurotoxicity of P407 nano-micelles after CED in vivo, we treated Wistar rats with intrastriatal infusion of nondrug-loaded P407/aCSF and analyzed their brains 72 hours and 3 weeks after treatment in comparison to the brains of animals treated with aCSF alone, and untreated animals. Brains were histologically examined specifically for the expression of the neuronal marker NeuN and glial protein GFAP by immunofluorescence. Juvenile animals should have relatively high levels of the presynaptic protein synaptophysin due to the increased amount of synaptogenesis in the developing brain. Significant synaptic toxicity and neuronal loss caused by infusion of P407 should therefore result in decreased levels of synaptophysin when compared to controls. Both toxicity assays did not show any difference between animals treated with P407/aCSF compared to aCSF-treated and untreated controls (Figure 5).

### Effect of acute CED of panobinostat-loaded nano-micelles on the survival of F98 glioma-bearing animals

**Figure 6:**
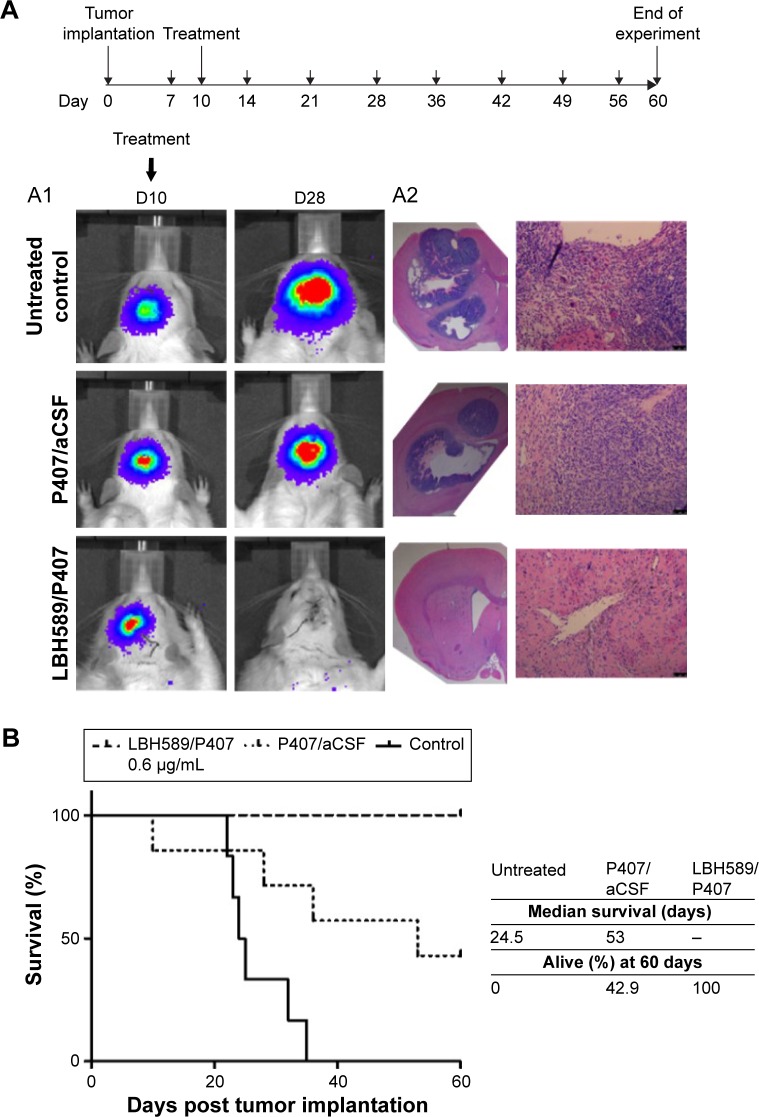
In vivo efficacy of LBH589/P407 after a single administration by CED in the syngeneic Fischer344/F98-Luc orthotopic rat glioma model. **Notes:** Animals with visible tumor bioluminescence were treated on day 10 after stereotactic implantation of 10^5^ luciferase-expressing F98 glioma cells into the left striatum (large arrows on schematic). All animals had weekly scans when alive (small arrows). (**A**) Bioluminescent imaging revealed a loss of transcranial bioluminescence in treated animals compared to controls (A1) and no evidence of tumor histologically post mortem or post sacrifice (A2 H&E). (**B**) Kaplan–Meier survival analysis of a 60-day experimental period showed a significant survival advantage in treated animals compared to untreated and vehicle-only controls (eight animals per group, log-rank test, *P*<0.001). All the treated animals (100%) survived. **Abbreviations:** CED, convection enhanced delivery; H&E, hematoxylin and eosin; P407, poloxamer 407.

Animals were randomly assigned to three groups of eight. Group 1 received 5 μL of LBH589/P407 at a concentration of 0.6 μg/mL administered by CED 10 days after tumor implantation. This dose was threefold higher than the IC_50_ for F98 cells in vitro after a 6-hour exposure to drug and was associated with an antitumor effect in vivo in our pilot experiments. Group 2 received P407/aCSF alone as a vehicle control, and group 3 received no treatment. All animals that received LBH589/P407 survived the experimental period and were in good health. Conversely, untreated animals and those treated with P407 alone did poorly. Kaplan–Meier survival analysis showed a significant effect on animal survival (log-rank test, *P*=0.0007) after a single treatment with LBH589/P407 compared to untreated control (Figure 6).

The brains of all animals underwent histological examination post mortem. All animals in the two control groups had large necrotic tumors visible on H&E-stained sections, including those that survived the experimental period. Conversely, animals that received treatment with LBH589/P407 did not have any evidence of microscopic tumor. All experimental animals had a transcranial bioluminescent signal 1 week after implantation and before treatment, indicating that this result is unlikely to be due to a failure in tumor grafting (Figure 6).

## Discussion

In this study, we showed that the pan-HDACi panobinostat was effective in prolonging the survival of glioma-bearing animals when administered in a water-soluble nano-micellar formulation by CED. The nano-micellar formulation was chosen as it provided a method by which an otherwise water-insoluble compound could be delivered in an aqueous solution that did not require the use of a potentially toxic solvent, which is important if this therapy is to translate to the clinic. Moreover, P407 nano-micelles formulated in aCSF were not toxic to neurons or glia in vitro and to rat brain when administered by CED in vivo when compared to animals that had been infused with aCSF alone after a 21-day interval. The polymer was intracellular within 15 minutes of incubation with glioma cells in vitro and distributed well in both white and gray matter after acute CED in vivo. Fluorescent polymer remained within the target structure for up to 48 hours after infusion.

The F98 rat glioma model was chosen because it is universally lethal, and it displays some of the histological hallmarks of HGG, namely high level of mitosis and necrosis, as well as weak immunogenicity. It therefore serves as a good model to test therapies that may have an immunotherapeutic effect.^40^ This was felt to be important, due to the observation that histone deacetylase (HDAC) inhibition may have immunoregulatory effects in other malignancies, such as Hodgkin’s lymphoma and myeloma, and their efficacy requires an intact immune system.^41^–^43^ HDAC inhibition may therefore be particularly effective in malignancies that are poorly immunogenic and in tumors that are associated with an immunosuppressive microenvironment, such as malignant glioma.^44^ Even though this effect of HDAC inhibition has yet to be demonstrated in HGG, we did not want to negate any potential immunotherapeutic effect of panobinostat delivered by CED by using an immunocompromised rat–human glioma xenograft. A rat rather than mouse model was used as the larger brain size is better suited to test therapies administered by CED. While we recognize the limitations of this model as an accurate representation of human HGG, it serves as a proof of concept that CED of LBH589/P407 nano-micelles is highly efficacious in vivo.

CED of LBH589/P407 nano-micelles was associated with 100% survival after a single infusion 10 days after tumor implantation, which was a dramatic and surprising result. The significant efficacy of LBH589/P407 nano-micelles demonstrated in this study is likely to be attributable to multiple factors including the higher free molecular concentration of panobinostat achieved by the solvent-mediated emulsion evaporation method. In addition, the observed rapid cellular uptake of P407 nano-micelles in vitro may increase the in vivo efficacy, and there may be a sustained release of insoluble drug payload from the micelle core over time. We also show that P407 remains in the target structure for up to 48 hours after infusion. We therefore hypothesize that the polymer may continue to release drug over this time as it biodegrades, which may also help to explain how a single infusion was so effective. Further investigations are required to prove a sustained release mechanism both in vitro and in vivo. In addition, the pharmacodynamics and pharmacokinetics of P407 after direct administration to the brain parenchyma are unknown and warrant further study. Bioaccumulation of polymer within the brain may be a concern if it was given repeatedly over time, and would be important to understand in a clinically applicable large animal model of intermittent CED before translation to the clinic.^45^,^46^

We also observed that CED of nondrug-loaded P407/aCSF nano-micelles was associated with a survival advantage (*P*=0.05; Figure 6). P407/aCSF nano-micelles were not cytotoxic to glioma cells in vitro. All of the animals treated with P407/aCSF had large necrotic tumors at the time of termination, even in those that survived the experimental period. This, in combination with the observation that P407/aCSF was not cytotoxic to glioma in vitro, suggests that this observed survival advantage is not likely to be due to the properties of the vehicle itself. We propose that the survival advantage seen in the vehicle control group may be due to local inflammation and mechanical damage to the tumor tissue caused by the infusion itself, rather than a drug effect. Despite this observation, the difference in survival between the treatment group and the vehicle control group was still significant (*P*=0.01).

## Conclusion

HGG carries a dismal prognosis and represents an unmet clinical need. Current therapies are ineffective, and the management of the disease requires a paradigm shift if we are going to improve the prognosis for affected patients. Direct intraparenchymal infusion of drug to affected regions of brain by CED may provide a valid therapeutic alternative for patients and has met with some success in early phase clinical trials and in reported cases.^5^,^7^,^13^–^15^,^47^ Drugs with preclinical promise often fail to reach clinical effect as they do not cross the BBB when given systemically. In addition, many new drugs are water insoluble, which limit their use in an aqueous formulation, as is required for CED. We have demonstrated a facile method for the delivery of poorly soluble drugs by CED in a glioma model with translational possibility. Panobinostat has the potential to have a significant impact on the treatment of HGG when delivered in a suitable formulation for CED and warrants rapid translation to the clinic.

## Supplementary materials

### Nuclear magnetic resonance (NMR) details

^1^H NMR experiments to investigate the behavior of the drug-loaded polymeric nano-micelles over time and upon dilution were conducted on a Varian 600 MHz VNMRS NMR Spectrometer fitted with a 5-mm HCN cold probe and using a double pulse field-gradient spin-echo water suppression pulse sequence.

As a control, 1.2 mg of as-provided bulk panobinostat was added to 1 mL of D_2_O, and the resultant suspension was moderately heated and shaken. Then, 450 μL was combined with 50 μL of a 2.4 mM standard solution of 4,4-dimethyl-4-silapentane-1-sulfonic acid sodium salt (DSS-Na) in D_2_O in an NMR tube. The integrals of the DSS-Na signal between 0.72 and 0.53 ppm (two protons) and the panobinostat signal between 7.22 and 7.03 ppm (two protons) were then recorded.

A second NMR sample containing 450 μL of a 0.4282 mg/mL (1.2 μM) solution of panobinostat in 5% poloxamer 407 (P407) artificial cerebrospinal fluid (aCSF) was combined with 50 μL of the same DSS-Na standard solution. The integrals of the DSS-Na signal between 0.66 and 0.58 ppm (two protons) and the panobinostat signal between 7.04 and 6.92 ppm (two protons) were then recorded.

The approximate amount of free molecular drug present in both samples could then be determined (precipitated solid fractions of the drug would not be detectable by NMR).

A third NMR sample containing 150 μL of a 0.4282 mg/mL (1.2 μM) solution of panobinostat in 5% P407 aCSF and 300 μL of pre-aCSF solution was prepared (ie, a 1:3 dilution). Then, 50 μL of the same DSS/D_2_O standard solution used in the control sample was added. Repeat NMR experiments on this diluted sample were carried out over a 24-hour period with no observable change in the level of free panobinostat in solution.

^1^H diffusion-ordered NMR spectroscopy (DOSY NMR) was performed on a Varian 500 MHz VNMRS NMR Spectrometer fitted with a 5-mm AutoX DB-PFG probe to confirm the presence and diffusion coefficient of free panobinostat.

### Synthesis of rhodamine-labeled pluronic P407 nano-micelles

Approximately 12.66 g of P407 was dissolved in dry acetonitrile (15 mL) and added dropwise to an excess amount, 1.62 g of *N*,*N*-carbonyldiimidazole (CDI) in dry acetonitrile (15 mL) at room temperature during a 2-hour period under nitrogen atmosphere. The resulting mixture was kept stirring for a further 2 hours before the removal of unreacted CDI by the addition of 0.2 mL of water. The neutralized solution was stirred for a further 20 minutes, then added dropwise over a 2-hour period to 10 mL of 1, 2-ethylenediamine at room temperature. The solution was allowed to react for 12 hours before the removal of excess 1,2-ethylenediamine by rotary evaporation to form a viscous transparent oil. This solution was transferred to a section of dialysis tubing MW, 3500 cutoff) and dialyzed against water for 5 days. The dialyzed solution was freeze-dried to isolate the P407-NH_2_ intermediate for use in the rhodamine cross-linking reaction.

Approximately 10 mL of dimethylformamide containing 0.323 g of P407-NH_2_ was degassed under nitrogen for 10 minutes before 67.5 mg of rhodamine isothiocyanate was dissolved into it. The reaction vial was wrapped in foil to prevent photobleaching of the rhodamine moiety and maintained under nitrogen for 12 hours to allow the rhodamine to couple to the pendant amine. The product solution was decanted into dialysis tubing (MW, 3500 cutoff) and dialyzed against a weak NaOH solution maintained at pH 8.0 for 7 days before the rhodamine-labeled P407 product was isolated by freeze-drying to form a bright purple product which was crystalline in appearance.

Figure S1 DLS number plot for P407 micelle solutions containing 0–1.44 mM concentration of panobinostat (LBH589).
**Abbreviations:** DLS, dynamic light scattering; P407, poloxamer 407.

Figure S2 Plot of a DOSY NMR spectrum showing the characteristic chemical shifts for panobinostat (LBH589) after water signal suppression in a 5% P407 solution synthesized using the emulsion evaporation method.
**Abbreviations:** DOSY NMR, diffusion-ordered nuclear magnetic resonance spectroscopy; P407, poloxamer 407.

Figure S3 A plot of diffusion coefficients for the various species of panobinostat (LBH589) present in solution.
**Notes:** The blue plot is the data taken from DLS of an LBH589/P407 micelle. The black lines represent the expected domain for small molecule species free in solution. The red lines indicate the range of diffusion coefficients observed for molecular LBH589 as calculated from NMR data.
**Abbreviations:** aCSF, artificial cerebrospinal fluid; DLS, dynamic light scattering; NMR, nuclear magnetic resonance; P407, poloxamer 407.

## References

[1] Stupp R, Mason WP, van den Bent MJ (2005). Radiotherapy plus concomitant and adjuvant temozolomide for glioblastoma. N Engl J Med.

[2] Bobo RH, Laske DW, Akbasak A, Morrison PF, Dedrick RL, Oldfield EH (1994). Convection-enhanced delivery of macromolecules in the brain. Proc Natl Acad Sci U S A.

[3] Morrison PF, Laske DW, Bobo H, Oldfield EH, Dedrick RL (1994). High-flow microinfusion: tissue penetration and pharmacodynamics. Am J Physiol.

[4] Panse SJ, Fillmore HL, Chen ZJ, Gillies GT, Broaddus WC (2010). A novel coaxial tube catheter for central nervous system infusions: performance characteristics in brain phantom gel. J Med Eng Technol.

[5] Kunwar S, Chang S, Westphal M (2010). Phase III randomized trial of CED of IL13-PE38QQR vs Gliadel wafers for recurrent glioblastoma. Neuro Oncol.

[6] Kunwar S, Prados MD, Chang SM (2007). Direct intracerebral delivery of cintredekin besudotox (IL13-PE38QQR) in recurrent malignant glioma: a report by the Cintredekin Besudotox Intraparenchymal Study Group. J Clin Oncol.

[7] Sampson JH, Akabani G, Archer GE (2008). Intracerebral infusion of an EGFR-targeted toxin in recurrent malignant brain tumors. Neuro Oncol.

[8] Gimenez F, Krauze MT, Valles F (2011). Image-guided convection-enhanced delivery of GDNF protein into monkey putamen. Neuroimage.

[9] Gill SS, Patel NK, Hotton GR (2003). Direct brain infusion of glial cell line-derived neurotrophic factor in Parkinson disease. Nat Med.

[10] Love S, Plaha P, Patel NK, Hotton GR, Brooks DJ, Gill SS (2005). Glial cell line-derived neurotrophic factor induces neuronal sprouting in human brain. Nat Med.

[11] Kells AP, Eberling J, Su X (2010). Regeneration of the MPTP-lesioned dopaminergic system after convection-enhanced delivery of AAV2-GDNF. J Neurosci.

[12] Barua NU, Gill SS, Love S (2014). Convection-enhanced drug delivery to the brain: therapeutic potential and neuropathological considerations. Brain Pathol.

[13] Barua NU, Lowis SP, Woolley M, O’Sullivan S, Harrison R, Gill SS (2013). Robot-guided convection-enhanced delivery of carbo-platin for advanced brainstem glioma. Acta Neurochir.

[14] Anderson RC, Kennedy B, Yanes CL (2013). Convection-enhanced delivery of topotecan into diffuse intrinsic brainstem tumors in children. J Neurosurg Pediatr.

[15] Bruce JN, Fine RL, Canoll P (2011). Regression of recurrent malignant gliomas with convection-enhanced delivery of topotecan. Neurosurgery.

[16] Falkenberg KJ, Johnstone RW (2014). Histone deacetylases and their inhibitors in cancer, neurological diseases and immune disorders. Nat Rev Drug Discov.

[17] Sturm D, Bender S, Jones DT (2014). Paediatric and adult glioblastoma: multiform (epi)genomic culprits emerge. Nat Rev Cancer.

[18] Zhang L, Chen LH, Wan H (2014). Exome sequencing identifies somatic gain-of-function PPM1D mutations in brainstem gliomas. Nat Genet.

[19] Grasso CS, Tang Y, Truffaux N (2015). Functionally defined therapeutic targets in diffuse intrinsic pontine glioma. Nat Med.

[20] Drappatz J, Lee EQ, Hammond S (2012). Phase I study of panobinostat in combination with bevacizumab for recurrent high-grade glioma. J Neurooncol.

[21] Lee EQ, Reardon DA, Schiff D (2015). Phase II study of panobinostat in combination with bevacizumab for recurrent glioblastoma and anaplastic glioma. Neuro Oncol.

[22] Rasmussen TA, Tolstrup M, Moller HJ (2015). Activation of latent human immunodeficiency virus by the histone deacetylase inhibitor panobinostat: a pilot study to assess effects on the central nervous system. Open Forum Infect Dis.

[23] Goldberg JM, Glade-Bender J, Sulis ML (2014). A phase I dose finding study of panobinostat in children with hematologic malignancies: initial report of TACL study T2009-012 in children with acute leukemia. Blood.

[24] Rodgers L, McCully CL, Peer C, Cruz R, Figg W (2016). HG-36 plasma and cerebrospinal fluid (csf) pharmacokinetics of panobinostat following oral administration to nonhuman primates. Neuro Oncol.

[25] Saito R, Krauze MT, Noble CO (2006). Tissue affinity of the infusate affects the distribution volume during convection-enhanced delivery into rodent brains: implications for local drug delivery. J Neurosci Methods.

[26] Ahmad Z, Shah A, Siddiq M, Kraatz H-B (2014). Polymeric micelles as drug delivery vehicles. RSC Adv.

[27] Kabanov AV, Batrakova EV, Miller DW (2003). Pluronic block copolymers as modulators of drug efflux transporter activity in the blood-brain barrier. Adv Drug Deliv Rev.

[28] Akash MS, Rehman K (2015). Recent progress in biomedical applications of Pluronic (PF127): pharmaceutical perspectives. J Control Release.

[29] Collins AM, Olof SN, Mitchels JM, Mann S (2009). Facile preparation and processing of aqueous dispersions of tris(8-hydroxyquinoline) aluminium(iii) photoluminescent nanoparticles. J Mater Chem.

[30] Collins AM, Zabkiewicz J, Ghiggi C, Hauser JC, Burnett AK, Mann S (2011). Tris(8-hydroxyquinolinato)gallium(III)-loaded copolymer micelles as cytotoxic nanoconstructs for cosolvent-free organometallic drug delivery. Small.

[31] Wanka G, Hoffmann H, Ulbricht W (1990). The aggregation behavior of poly-(oxyethylene)-poly-(oxypropylene)-poly-(oxyethylene)-block-copoly-mers in aqueous solution. Colloid Polym Sci.

[32] Lu HF, Lim WS, Wang J (2003). Galactosylated PVDF membrane promotes hepatocyte attachment and functional maintenance. Biomaterials.

[33] Zhang W, Shi Y, Chen Y (2010). Enhanced antitumor efficacy by paclitaxel-loaded pluronic P123/F127 mixed micelles against non-small cell lung cancer based on passive tumor targeting and modulation of drug resistance. Eur J Pharm Biopharm.

[34] Pollard SM, Yoshikawa K, Clarke ID (2009). Glioma stem cell lines expanded in adherent culture have tumor-specific phenotypes and are suitable for chemical and genetic screens. Cell Stem Cell.

[35] Stricker SH, Feber A, Engstrom PG (2013). Widespread resetting of DNA methylation in glioblastoma-initiating cells suppresses malignant cellular behavior in a lineage-dependent manner. Genes Dev.

[36] Arshad A, Yang B, Bienemann AS (2015). Convection-enhanced delivery of carboplatin PLGA nanoparticles for the treatment of glioblastoma. PLoS One.

[37] Siew LK, Love S, Dawbarn D, Wilcock GK, Allen SJ (2004). Measurement of pre- and post-synaptic proteins in cerebral cortex: effects of postmortem delay. J Neurosci Methods.

[38] Taylor H, Barua N, Bienemann A (2013). Clearance and toxicity of recombinant methionyl human glial cell line-derived neurotrophic factor (r-metHu GDNF) following acute convection-enhanced delivery into the striatum. PLoS One.

[39] Bryant MJ, Chuah TL, Luff J, Lavin MF, Walker DG (2008). A novel rat model for glioblastoma multiforme using a bioluminescent F98 cell line. J Clin Neurosci.

[40] Barth RF, Kaur B (2009). Rat brain tumor models in experimental neuro-oncology: the C6, 9L, T9, RG2, F98, BT4C, RT-2 and CNS-1 gliomas. J Neurooncol.

[41] Oki Y, Buglio D, Zhang J (2014). Immune regulatory effects of panobinostat in patients with Hodgkin lymphoma through modulation of serum cytokine levels and T-cell PD1 expression. Blood Cancer J.

[42] Woods DM, Woan K, Cheng F (2013). The antimelanoma activity of the histone deacetylase inhibitor panobinostat (LBH589) is mediated by direct tumor cytotoxicity and increased tumor immunogenicity. Melanoma Res.

[43] West AC, Mattarollo SR, Shortt J (2013). An intact immune system is required for the anticancer activities of histone deacetylase inhibitors. Cancer Res.

[44] Jackson C, Ruzevick J, Phallen J, Belcaid Z, Lim M (2011). Challenges in immunotherapy presented by the glioblastoma multiforme microenvironment. Clin Dev Immunol.

[45] Barua NU, Woolley M, Bienemann AS (2013). Intermittent convection-enhanced delivery to the brain through a novel transcutaneous bone-anchored port. J Neurosci Methods.

[46] White E, Woolley M, Bienemann A (2011). A robust MRI-compatible system to facilitate highly accurate stereotactic administration of therapeutic agents to targets within the brain of a large animal model. J Neurosci Methods.

[47] Barua NU, Hopkins K, Woolley M (2016). A novel implantable catheter system with transcutaneous port for intermittent convection-enhanced delivery of carboplatin for recurrent glioblastoma. Drug Deliv.

