# Supplementary figures and images for: Convection enhanced delivery of panobinostat (LBH589)-loaded pluronic nano-micelles prolongs survival in the F98 rat glioma model
Source: Int J Nanomedicine. 2017 Feb 21;12:1385–99. doi: 10.2147/IJN.S125300 (PMC5327904; doi:10.2147/IJN.S125300)

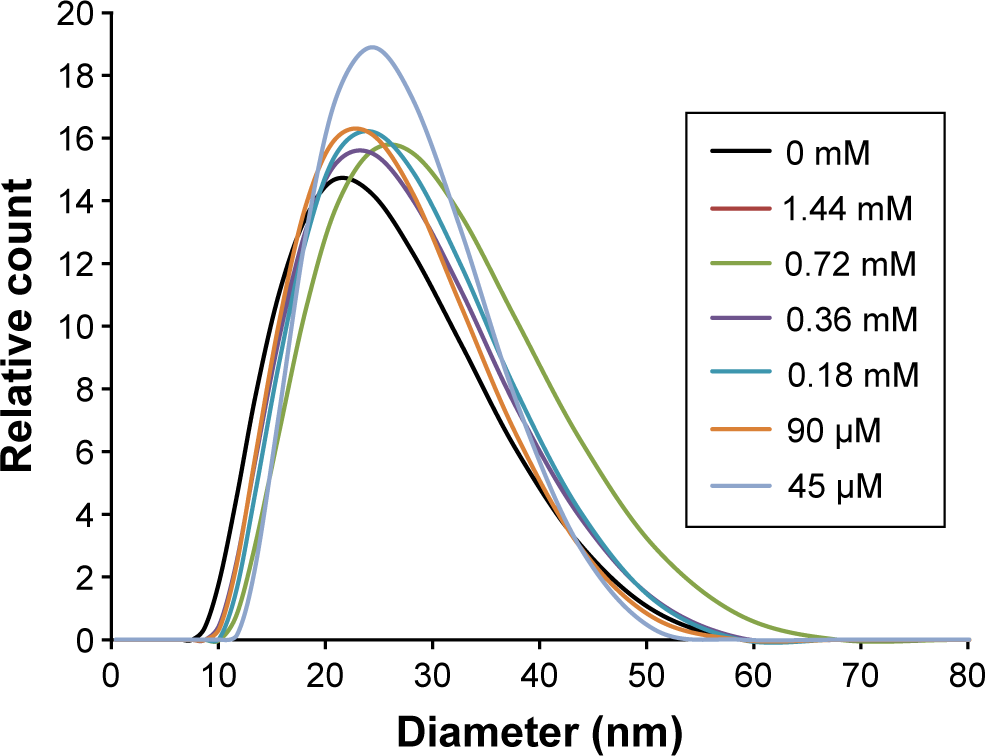

Supplement: Figure S1 — DLS number plot for P407 micelle solutions containing 0–1.44 mM concentration of panobinostat (LBH589). Abbreviations: DLS, dynamic light scattering; P407, poloxamer 407. [file ijn-12-1385s1.tif]

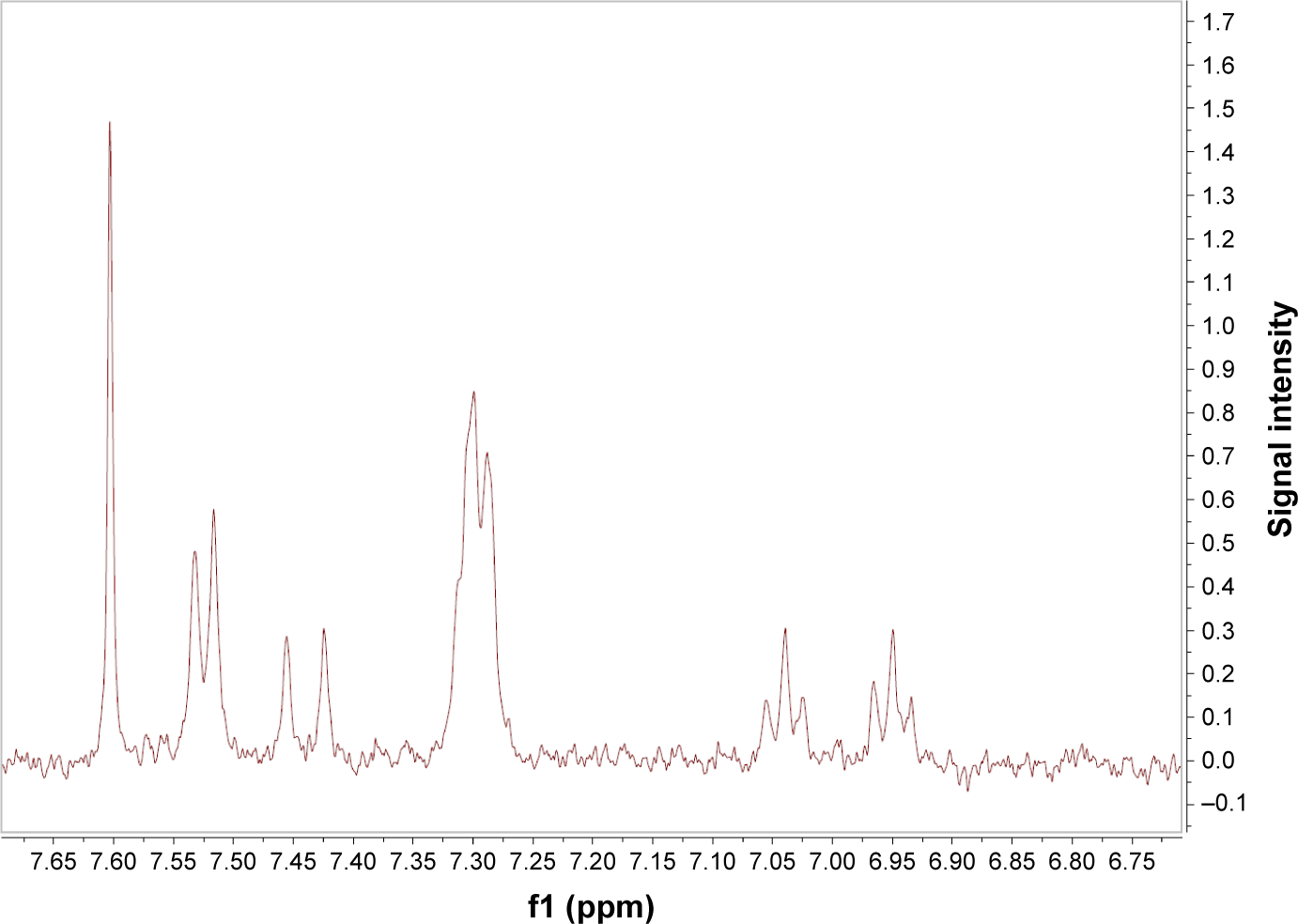

Supplement: Figure S2 — Plot of a DOSY NMR spectrum showing the characteristic chemical shifts for panobinostat (LBH589) after water signal suppression in a 5% P407 solution synthesized using the emulsion evaporation method. Abbreviations: DOSY NMR, diffusion-ordered nuclear magnetic resonance spectroscopy; P407, poloxamer 407. [file ijn-12-1385s2.tif]

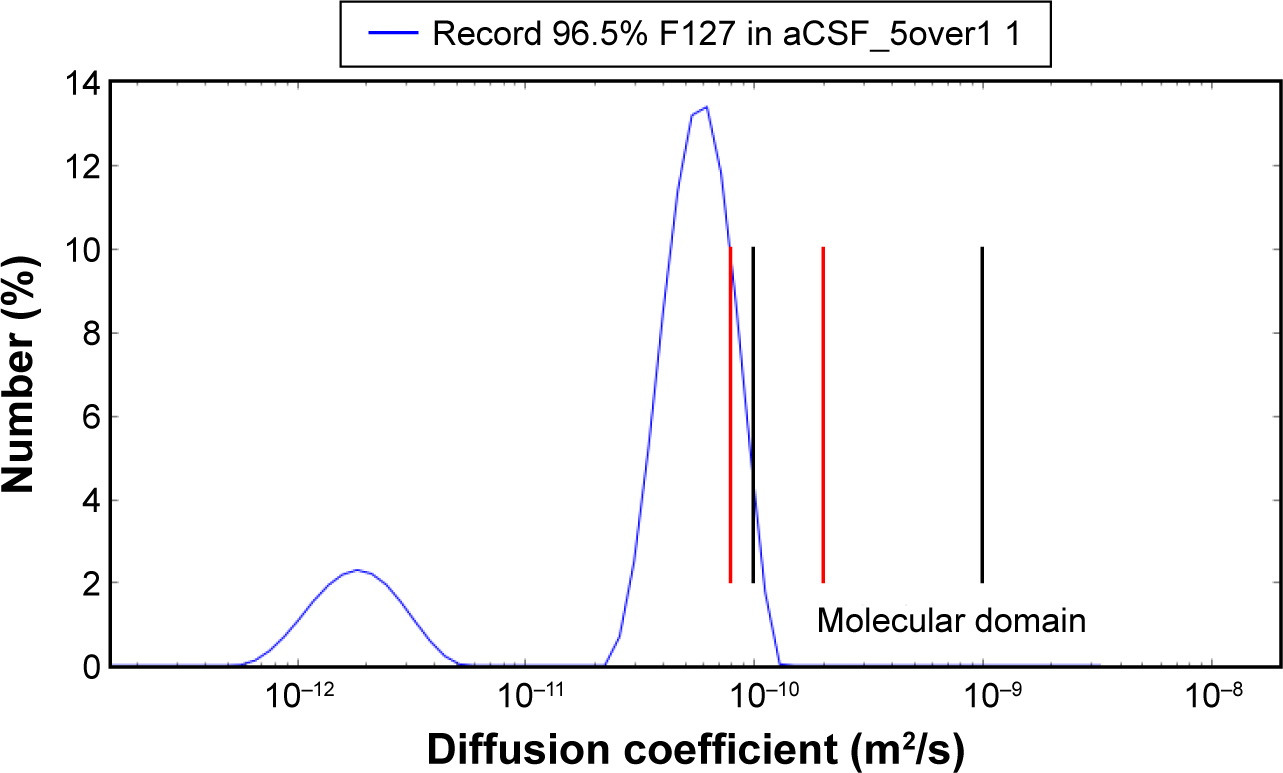

Supplement: Figure S3 — A plot of diffusion coefficients for the various species of panobinostat (LBH589) present in solution. Notes: The blue plot is the data taken from DLS of an LBH589/P407 micelle. The black lines represent the expected domain for small molecule species free in solution. The red lines indicate the range of diffusion coefficients observed for molecular LBH589 as calculated from NMR data. Abbreviations: aCSF, artificial cerebrospinal fluid; DLS, dynamic light scattering; NMR, nuclear magnetic resonance; P407, poloxamer 407. [file ijn-12-1385s3.tif]
